# Tremor-Ataxia syndrome and primary ovarian insufficiency in an***FMR1*** premutation carrier

**DOI:** 10.25100/cm.v48i3.3019

**Published:** 2017-09-30

**Authors:** Wilmar Saldarriaga-Gil, Tatiana Rodriguez-Guerrero, Andres Fandiño-Losada, Julian Ramirez-Cheyne

**Affiliations:** 1 Grupo de investigación MACOS, Departamento Morfología, Facultad de Salud Universidad del Valle, Cali, Colombia.; 2 Departamento de Medicina y Cirugía, Facultad de Salud, Universidad del Valle, Cali, Colombia.; 3 Escuela de Salud Pública, Universidad del Valle, Cali, Colombia.

**Keywords:** Fragile X Tremor Ataxia Syndrome, Ataxia/complications, Ataxia/diagnosis, Ataxia/genetics, FXPOI, FXTAS, *FMR1*gen, síndrome de temblor y ataxia X fragil, complicaciones/ataxia, diagnostico/ataxia, genetica/ataxia, FXPOI, FXTAS, gen *FMR1*

## Abstract

**Introduction:**

The *FMR1* gene has four allelic variants according to the number of repeats of the CGG triplet. Premutation carriers with between 55 and 200 repeats are susceptible to developing pathologies such as tremor and ataxia syndrome (FXTAS) and fragile X-associated primary ovarian insufficiency (FXPOI) syndrome.

**Case description:**

The patient was a 53-year-old female farmer with severe tremor in the upper limbs at rest that worsens with movement, tremor in the jaw and tongue, and generalized cerebral atrophy. She is a carrier of the *FMR1* premutation diagnosed by PCR and Southern Blot, complying with the clinical and radiological criteria of FXTAS, and in addition, has a history of vagal symptoms suggestive of ovarian failure and menstrual cycle disorders that led to hysterectomy at age 33 and was subsequently diagnosed with FXPOI.

**Conclusion:**

An unusual case of FXTAS and FXPOI complying with clinical and radiological criteria is reported in a premutation carrier of the *FMR1* gene*.*

## Resumen

**Introducción:**

el gen *FMR1* tiene cuatro variantes alélicas según el número de repeticiones de la tripleta CGG. Los portadores de la premutación con un número entre 55 y 200 repeticiones son susceptibles de desarrollar patologías como el síndrome de temblor y ataxia (FXTAS) y síndrome de falla ovárica prematura (FXPOI) asociados al X frágil.

**Descripción del caso:**

Mujer de 53 años, agricultora, con temblor severo en miembros superiores en reposo que empeora con el movimiento, temblor en mandíbula y lengua, atrofia cerebral generalizada, portadora de la premutación del gen *FMR1* diagnosticada por PCR y Southern Blot, cumpliendo con criterios clínicos y radiológicos de FXTAS; ademas, historia de síntomas vagales sugestivos de falla ovárica y trastornos del ciclo menstrual que llevaron a histerectomía a los 33 años, haciendose diagnóstico FXPOI.

**Conclusión:**

Se reporta un caso inusual en portadoras de la premutación del gen *FMR1,* con criterios clínicos y radiológicos de FXTAS y FXPOI.

## Introduction

The FMR1 (Fragile X Mental Retardation 1 Gene) gene, with locus Xq27.3, has 17 exons spanning 38 kb, OMIM * 309550 1. This gene is characterized by presenting variable numbers of nucleotide triplet repeats of cytosine-guanine-guanine (CGG) in the 5' untranslated region (UTR5'). According to the number of triplets, four allelic variants occur: full mutation (MC) (>200 triplets), premutated (PM) (55-200), gray area (ZG) (45-54), and normal (<45) 2.

The carriers of the premutation have a variable phenotype and incomplete penetrance completely different than those who have CM and are affected by Fragile X syndrome (FXS) 3,4.

Pathologies found in carriers have been referred to as FRAXopathies, and the most well-known are tremor and ataxia associated with FXS (Fragile X Tremor Ataxia Syndrome; FXTAS) and primary ovarian insufficiency syndrome associated with FXS (FXPOI) 5. The diagnosis of these entities is made mainly through clinical findings and specific signs in nuclear magnetic resonance (MRI) of the brain 3,5. Environmental agents have been associated with the early onset and greater severity of symptoms 3,4.

The objective of this article is to contribute to the literature by reporting a rare case of FXTAS and FXPOI in a carrier of FMR1 premutation with the appearance of symptoms at an early age and severe symptomatology. This is the first report of this association in Colombia.

## Case description

The patient is originally from a Colombian population north of Valle del Cauca, a region with a high prevalence of premutation and full mutation of the FMR1 gene.

**Figure 1:**
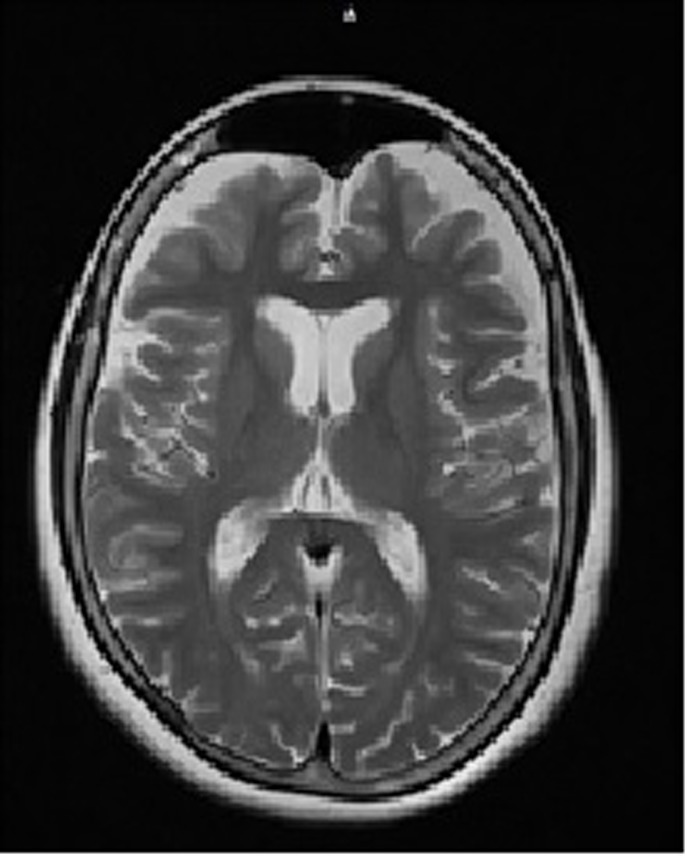
Magnetic resonance of the brain with gadolinium. Contrast-enhanced brain MRI shows marked cortical and subcortical atrophy in the frontal region plus dilation of the ventricular system.

The patient is a 53-year-old woman, a farmer, with a 4-year history of distal tremor at rest that worsens with movement, with a greater involvement of the upper limbs, that has become increasingly severe. In the last year, the tremor involved the mandibular region and the tongue. Therefore, the patient repeatedly consulted a physician; electrolyte and thyroid function studies were performed, and cerebral structural alterations were measured by computerized axial tomography. All results were within normal limits. However, due to the tremor's worsening, a contrast-enhanced brain MRI was performed and showed significant cortical and subcortical atrophy, with greater frontotemporal involvement, and an increase in the size of the ventricular system, markedly in the third ventricle Fig. 1. Other findings were not significant.

Subsequently, a molecular study was performed for the quantification of FMR1 gene triplets with PCR using a triple primer, including a CGG-specific primer and Southern Blot. Thirty and 82 repeats were found in each allele. 

Personal pathological antecedents included convulsive syndrome in childhood that was spontaneously resolved at 14 years old; medically managed bilateral carpal tunnel; a gestational formula G5P3C2V2Mo3; and irregular menstrual cycles characterized by persistent polymenorrhea and metrorrhagia, without improvement despite medical management that forced a hysterectomy at the age of 33 years. The living children were studied, and none had PM or MC of the FMR1 gene. In the year prior to hysterectomy and up to 2 years later, she reported perimenopausal symptoms such as hot flashes, profuse night sweats, persistent headache, and vaginal dryness. 

At the time of the physical examination, there was a tremor at rest that involved the upper limbs, the jaw and the tongue, which worsened during activity. In spite of the limitation for performing cerebellar tests imposed by the tremor, no alterations in gait pattern or other signs of cerebellar ataxia were documented. The Fahn-Tolosa-Marín tremor rating scale 6 was applied to obtain an objective approximation to the findings of the physical examination, and a 15% severity of the involvement was measured Table 1.

Another relevant element found in the physical examination was the presence of neuropathy in the four extremities caused by the alteration of superficial sensitivity with the presence of hypoesthesia and paresthesia.

During the interview, manifestations of anxiety with affective modulation were observed. The GAD-7 Scale 7 was then applied with a result that suggested a moderate generalized anxiety disorder. 

In addition, an evaluation of the patient's cognitive status was Fahn-Tolosa-Marín performed through the Mini-Mental test in which 25 points out of 30 were obtained, which is suggestive of a pathology.

**Table 1:** Fahn-Tolosa-Marín tremor rating scale applied to the patient.

Location of tremor	Lips	Jaw	Upper limbs
Classification	Resting	Resting	Resting and Postural
Assessment of tremor in upper limbs			
Intensity	Marked	Marked	Marked
Writing	Minimally abnormal		
When outlining	Moderate or crosses lines frequently		
When pouring	More careful than a person without tremors, but without spilling water		
Functional disability that causes tremor			
When talking	No		
Eating	Slightly abnormal; can bring food to the mouth and only spills rarely		
Bring liquids to the mouth	Slightly abnormal; can use the spoon, but not if it is completely full		
Hygiene	Slightly abnormal; does everything, but carefully		
Dress	Slightly abnormal; able to do everything, but carefully		
Write	Slightly abnormal; legible, able to write long letters		
Work	Does not interfere with work		
Overall rating by the examiner	Moderate disability (25-49%)		
Overall rating by the patient	Mild disability (1-24%)		
Total score			15%

The testing was assessed by a neurologist who initiated treatment with propanolol for tremor management and also by a psychiatrist who considered psychotherapeutic management without the need for medication at that time. 

## Discussion

The prevalence of FMR1 premutation is 11.7 per 10,000 males (95% CI: 6.0-18.7) and 34.4 per 10,000 females (95% CI: 6.3-83.3), corresponding to 1 for every 855 males and 1 for every 291 females 8,9. There are no data on the prevalence of allelic variants of the FMR1 gene in Columbia. 

The carriers of the premutation can develop pathologies called FRAXopathy, such as FXTAS and FXPOI 2. In addition, adults with and without FXTAS or FXPOI are at greater risk than the general population for presenting with affective problems, depression, anxiety, obsessive compulsive disorder, sleep disturbances, sleep apnea, neuropathies, psychiatric disorders, hypertension, migraine, fibromyalgia, and disorders of the thyroid gland 3,5,10-12. Additionally, in childhood, greater risk of anxiety, attention deficit and hyperactivity, autistic spectrum disorders and seizures can be observed in carriers 3, 13.

The phenotype of those affected by the MC of the FMR1 gene is completely different and does not have the classic features of FXS such as mild to moderate intellectual disability (ID), autistic spectrum disorders, elongated face, prognathism, large winged ears, joint hypermobility, macroorchidism, language deficit, anxiety and aggression. In females, the phenotype is usually less severe 11.

FXPOI is found in 20% of premutation carriers. Menstrual bleeding often stops for a year before the age of 40, which is a diagnosis of primary ovarian insufficiency. This frequency is increased 20-fold when compared to the general population, where primary ovarian insufficiency is approximately 1% 14. In addition, ovarian failure has been reported to be higher in carriers at all ages at which studies have been performed: 1.4% at age 18 years, and 3.0% at age 29 years versus 1 in 1,000 in the general population; in addition, carriers have menopause at an early age after 40 years 14. In these females, there are other signs and secondary symptoms such as hormonal alterations with elevated levels of FSH and low levels of estradiol, irregular menstrual cycles, perimenopausal symptoms and decreased fertility 2, 8, 15, 16. 

In the patient reported here, the association of irregular menstrual cycles that led to hysterectomy at age 33, vagal symptoms (related to menopause) and the finding of FMR1 premutation led us to conclude that the patient had primary ovarian insufficiency and FXPOI.

FXTAS is a progressive neurodegenerative disorder characterized by neurological deficits that include progressive intention tremor, cerebellar ataxia, cognitive deficit, parkinsonism, neuropathies, autonomic dysfunction and dementia. This syndrome occurs in 40% of male carriers at age 60 and in 75% at age 80 and occurs in 20% of females 3, 4, 9. In the general population, the estimated prevalence is 1 in 4,000 males older than 55 years and 1 in 7,800 in females 3.

In FXTAS, the alteration in the FMR1 gene does not silence the FMR1 gene as in those affected by the FXS in which the FMRP (Fragile X Mental Retardation Protein) is diminished or absent 3,17. On the contrary, there is overexpression, producing between 2-8 times the normal amount of FMR1 mRNA 2,3,4. The latter would be responsible for neuronal toxicity and lead to late presentation of neurodegenerative disorders 4. In FXPOI, it has not been possible to demonstrate how the mRNA level affects the hypothalamus-pituitary-gonad axis or the ovary directly 16. 

The diagnosis of FXTAS in FMR1 premutation carriers combines clinical, radiological, neuroimaging and pathological criteria 12. The major criteria are intention tremor, cerebellar ataxia, cognitive impairment and hyperintensity of the middle cerebellar peduncle, and rounded intranuclear inclusions typical of FXTAS in neurons and astrocytes. Minor criteria include short-term memory problems of moderate to severe intensity, neuropathy, deficits in executive functions, parkinsonism, generalized cerebral atrophy, and white matter disease in the brain or cerebellum. The symptoms are less severe in females 3,4, 18.

For a definitive diagnosis of FXTAS, it is necessary to have two major criteria: a major clinical criterion plus a major radiological criterion or pathological criterion; the presence of two major clinical criteria or a minor clinical criterion plus a minor radiological criterion make a probable diagnosis. Finally, a possible diagnosis of FXTAS is based on the presence of a minor clinical criterion and a minor clinical criterion 3,4.

The diagnosis of FXTAS in the patient reported here was made by having two major clinical criteria: intension tremor of severe intensity and sustained cognitive impairment demonstrated by a lower than expected score in the Mini-Mental test, and the minor radiological criterion being generalized and marked cortical atrophy in the frontal region. In addition, generalized anxiety was noted, and neuropathy was present as carpal tunnel syndrome. The severity of the symptoms and the early age of the FXTAS make the case infrequent and of poor prognosis.

However, in this patient, the age of onset of FXPOI at approximately 33 years, the age of onset of FXTAS symptoms before age 50, the severity of symptoms such as intention tremor that prevents her from performing activities of daily living, mandibular tremor, cognitive deficits, anxiety and generalized cortical atrophy make us consider that this is a very rare case, not only because of the low prevalence of FXTAS in females but also because of the poor presentation of FXTAS and FXPOI simultaneously, with a natural history of unusual disease. 

Treatment of FXTAS is mainly symptomatic, with therapeutic options that seek to alleviate neuromotor and psychiatric symptoms, with beta blockers, such as propanolol, being the most used for the relief of essential tremor, followed by anticonvulsants. Patients with ataxia have shown improvement with the use of memantine 19. Selective serotonin reuptake inhibitors (SSRIs) indicated in many of the mixed disorders associated with anxiety or depression are useful in patients with FXTAS. Physical and recreational activities and folate and vitamin B12 supplementation are used to maintain motor and cognitive abilities and reduce brain atrophy 3,19. Descriptions of the FRAXopathies 4 have been relatively recent and access to molecular tests is limited in our environment. We suggest that patients with primary ovarian insufficiency, tremor and/or ataxia should be suspected of having premutation of the FMR1 gene. The probability of diagnosis is increased when there is a specific family history of FXS or intellectual disability of unknown origin and also when specific criteria are found in brain images. The diagnosis should be discarded or confirmed with molecular tests such as PCR and Southern Blot specific for the FMR1 gene 2.

## Conclusions

We present a patient with clinical and radiological criteria indicating FXTAS and FXPOI with the appearance of symptoms at an early age and severe symptomatology, showing a natural history of the rare disease in carriers of FMR1 gene premutation.

## References

[1] Online Mendelian Inheritance in Man An Online Catalog of Human Genes and Genetic Disorders. Entry - # 300624 - Fragile X Mental Retardation Syndrome.

[2] Saldarriaga W, Tassone F, González-Teshima LY, Forero-Forero JV, Ayala-Zapata S, Hagerman R (2014). Síndrome de X Frágil. Colomb Med (Cali).

[3] Hagerman RJ, Hagerman P (2016). Fragile X-associated tremor/ataxia syndrome - features, mechanisms and management. Nat Rev Neurol.

[4] Pirozzi F, Tabolacci E, Neri G (2011). The FRAXopathies: Definition, overview, and update. Am J Med Genet A.

[5] Saldarriaga W, Lein P, González TLY, Isaza C, Rosa L, Polyak A (2016). Phenobarbital use and neurological problems in FMR1 premutation carriers. Neurotoxicology.

[6] Faher S, Tolosa E, Marin C, Jankovic J, Tolosa E (1988). Clinical rating scale for tremor. Parkinson´s Disease and Movement Disorder.

[7] Spitzer RL, Kroenke K, Williams JBW, Löwe B (2006). A brief measure for assessing generalized anxiety disorder The GAD-7. Arch Internal Med.

[8] Hunter J, Rivero-Arias O, Angelov A, Kim E, Fotheringham I, Leal J (2014). Epidemiology of fragile X syndrome A systematic review and meta-analysis. Am J Med Genet A.

[9] Grigsby J, Brega AG, Bennett RE, Bourgeois JA, Seritan AL, Goodrich GK (2016). Clinically significant psychiatric symptoms among male carriers of the fragile X premutation, with and without FXTAS, and the mediating influence of executive functioning. Clin Neuropsychol.

[10] Cordeiro L, Abucayan F, Hagerman R, Tassone F, Hessl D (2015). Anxiety disorders in fragile X premutation carriers Preliminary characterization of probands and non-probands. Intractable Rare Dis Res.

[11] Grigsby J, Brega AG, Bennett RE, Bourgeois JA, Seritan AL, Goodrich GK (2016). Clinically significant psychiatric symptoms among male carriers of the fragile X premutation, with and without FXTAS, and the mediating influence of executive functioning. Clin Neuropsychol.

[12] Lozano R, Saito N, Reed D, Eldeeb M, Schneider A, Hessl D (2016). Aging in Fragile X Premutation Carriers. Cerebellum.

[13] Chonchaiya W, Au J, Schneider A, Hessl D, Harris SW, Laird M (2012). Increased prevalence of seizures in boys who were probands with the FMR1 premutation and co-morbid autism spectrum disorder. Hum Genet.

[14] Welt CK, Smith PC, Taylor AE (2004). Evidence of early ovarian aging in fragile X premutation carriers. J Clin Endocrinol Metab.

[15] Wheeler AC, Raspa M, Green A, Bishop E, Bann C, Edwards A (2014). Health and reproductive experiences of women with an FMR1 premutation with and without fragile X premature ovarian insufficiency. Front Genet.

[16] Sherman SL, Allen EG, Spencer JB, Nelson LM, Tassone F, Hall DA (2016). Clinical manifestation and management of FXPOI. FXTAS, FXPOI, and Other Premutation Disorders.

[17] Sethna F, Moon C, Wang H (2014). From FMRP function to potential therapies for fragile X syndrome. Neurochem Res.

[18] Usdin K, Hukema RK, Sherman SL, Tassone F, Hall DA (2016). Model systems for understanding FXPOI. FXTAS, FXPOI, and Other Premutation Disorders.

[19] Hall DA, Berry-Kravis E, Hagerman RJ, Hager- Man PJ, Rice CD, Leehey MA (2006). Symptomatic treatment in the fragile X-associated tremor/ataxia syndrome. Mov Disord.

