# Supplementary material for: Tremor-Ataxia syndrome and primary ovarian insufficiency in anFMR1 premutation carrier
Source: Colomb Med (Cali). 2017 Sep 30;48(3):148–51. doi: 10.25100/cm.v48i3.3019 (PMC5687867; doi:10.25100/cm.v48i3.3019)
Supplement: Translation

# Translation: Síndrome de Tremor Ataxia y falla ovárica prematura en portadora de la premutación del gen ***FMR1***

## Introducción

El gen FMR1 (Fragile X Mental Retardation 1 Gene), con locus Xq27.3, tiene 17 exones que abarcan 38 Kb, OMIM *309550 1; este gen se caracteriza por presentar en la región no traducida 5´ (UTR5´), números variables de repeticiones de la tripleta de nucleótidos Citocina Guanina Guanina CGG; según el número de tripletas se producen cuatro variantes alélicas: mutación completa (MC) (>200), premutación (PM) (55-200), zona gris (ZG) (45-54), normal (<45) 2.

Los portadores de la premutación tienen un fenotipo variable y de penetrancia incompleta completamente diferente a quienes tienen la MC y son afectados por el síndrome X Frágil (SXF) 3,4.

A las patologías encontradas en portadores se les ha denominado FRAXOPATIAS y las más conocidas son el síndrome de temblor y ataxia asociado al SXF (FXTAS, abreviado de las palabras en inglés Fragile X Tremor Ataxia Syndrome) y el síndrome de falla ovárica primaria asociado al SXF (FXPOI) 5. El diagnostico de estas entidades se hace principalmente a través de hallazgos clínicos y signos específicos en resonancia nuclear magnética (RMN) de cerebro 3,5. Agentes ambientales se han asociado con la aparición temprana y mayor severidad de los síntomas 3,4.

 El objetivo de este artículo es aportar a la literatura un raro caso de FXTAS y FXPOI en una portadora de la premutación del gen FMR1, con aparición de los síntomas a edad temprana y sintomatología severa, siendo este el primer reporte de esta asociación en Colombia.

## Descripción del caso

La paciente es originaria y procedente de una población Colombiana al norte del Valle del Cauca, región con elevada prevalencia de premutación y mutación completa del gen FMR1.

Mujer de 53 años, agricultora, con una historia de 4 años de temblor distal en reposo que empeora con el movimiento, con un mayor compromiso de los miembros superiores, el cual se hace cada vez más severo; en el último año, el temblor comprometió la región mandibular y la lengua. Por lo anterior, ella consultó en repetidas ocasiones y se realizaron estudios de electrolitos, función tiroidea y alteraciones estructurales cerebrales mediante una tomografía axial computarizada, todos los resultados dentro de los límites normales. Sin embargo, por empeoramiento del temblor le realizaron RMN contrastada de cerebro, la cual mostró una atrofia cortical y subcortical significativa, con un mayor compromiso frontotemporal, un aumento del tamaño del sistema ventricular, marcado en el tercer ventrículo; sin otros hallazgos Fig. 1.

**Figura 1:** Resonancia Magnética de Cerebro con gadolinio. La RMN de cerebro con contraste muestra atrofia cortical y subcortical marcada en la región frontal más dilatación del sistema ventricular.

Posteriormente, se le realizó un estudio molecular para la cuantificacion de las tripletas del gen FMR1 con PCR, usando un triple cebador, incluyendo un cebador especifico para CGG y Southern Blot. Se encontraron 30 y 82 repeticiones en cada alelo. 

Con los antecedentes patológicos personales que incluyeron: síndrome convulsivo en la infancia que se resolvió espontáneamente a los 14 de años, túnel de carpo bilateral en manejo médico; una fórmula gestacional G5P3C2V2Mo3; los ciclos menstruales irregulares caracterizados por polimenorreas y metrorragias persistentes, sin mejoría a pesar del manejo médico, que obligaron a la realización de una histerectomía a la edad de 33 años. Los hijos vivos fueron estudiados y ninguno presentó PM o MC del gen FMR1. Refirió, en el año previo a la histerectomía y hasta dos años después, síntomas perimenopáusicos como oleadas de calor, sudoración nocturna profusa, cefalea persistente y sequedad vaginal. 

Al momento del examen físico se evidenció temblor en reposo que compromete los miembros superiores, la mandíbula y la lengua, que empeora durante la actividad; a pesar de la limitación para la realización de pruebas cerebelosas impuestas por el temblor, no se documentaron alteraciones del patrón de marcha, ni otros signos de ataxia cerebelosa. Buscando una aproximación objetiva a los hallazgos del examen físico se aplicó la Escala de Temblor de Fahn, Tolosa y Marín 6, obteniendo un 15% de severidad del compromiso Tabla 1.

**Tabla 1:** Escala de Temblor de Fahn, Tolosa y Marín aplicada a la paciente.

Localización del temblor	Labios	Mandíbula	Miembros superiores
Clasificación	Reposo	Reposo	Reposo y Postural
Valoración del temblor en miembros superiores			
Intensidad	Marcada	Marcada	Marcada
Escritura	Mínimamente anormal		
Al delinear	Moderado o cruza líneas frecuentemente		
Al verter	Más cuidadoso que una persona sin temblor, pero sin derramar agua		
Incapacidad funcional que genera el temblor			
Al hablar	No		
Al comer	Levemente anormal. Puede llevar los alimentos a la boca y sólo los derrama raramente		
Llevar líquidos a la boca	Levemente anormal. Puede usar la cuchara, pero no si está completamente llena		
Higiene	Levemente anormal. Lo realiza todo, pero cuidadosamente		
Vestirse	Levemente anormal. Capaz de hacerlo todo, pero de una forma cuidadosa		
Escribir	Levemente anormal. Legible, capaz de escribir cartas largas		
Trabajo	No interfiere con el trabajo		
Valoración global del examinador	Incapacidad moderada (25-49%)		
Valoración global del paciente	Incapacidad leve (1-24%)		
Puntaje total			15%	

Otro elemento relevante encontrado en el examen físico fue la presencia de una neuropatía en las cuatro extremidades, causada por la alteración de la sensibilidad superficial, con la presencia de hipoestesia y parestesias.

Durante la entrevista con modulación del afecto, se observaron manifestaciones de ansiedad. Se aplicó entonces, la Escala GAD-7 7 con un resultado que sugirió un trastorno de ansiedad generalizada moderado. 

Además, se realizó una evaluación del estado cognitivo de la paciente a través del test de MINIMENTAL, en el cual se obtuvieron 25 puntos de 30 posibles, siendo este resultado sugestivo de una patología.

Fue valorada por neurología quien inició un tratamiento con propanolol para manejo del temblor y también por psiquiatría quien consideró un manejo psicoterapéutico sin necesidad de medicación en ese momento. 

## Discusión

La prevalencia de la premutación del gen FMR1 es de 11.7 por cada 10,000 hombres (IC 95%: 6.0-18.7) y 34.4 por cada 10,000 mujeres (IC 95%: 6.3-83.3), correspondiendo a 1 por cada 855 hombres y 1 por cada 291 mujeres 8,9. En Colombia no hay datos de la prevalencia de las variantes alélicas del gen FMR1. 

Los portadores de la premutación pueden desarrollar patologías denominadas FRAXOPATIAS, como FXTAS y FXPOI 2. Además, los adultos con y sin FXTAS o FXPOI presentan más riesgo que la población general, de presentar problemas afectivos, depresión, ansiedad, trastorno obsesivo compulsivo, alteraciones del sueño, apnea del sueño, neuropatías, trastornos psiquiátricos, hipertensión, migraña, fibromialgia, y trastornos de la glándula tiroides 3,5,10-12. Igualmente en la infancia se puede observar ansiedad, déficit de atención e hiperactividad, trastornos del espectro autista y convulsiones (3, 13).

El fenotipo de los afectados por la MC del gen FMR1 es completamente diferente y no tienen las características clásicas del SXF como discapacidad intelectual (DI) leve a moderada, trastornos del espectro autista, cara alargada, prognatismo, orejas grandes y aladas, articulaciones hiperextensibles, macroorquidismo, déficit del lenguaje, ansiedad y agresión; en mujeres el fenotipo suele ser menos severo 11.

El FXPOI se encuentra en el 20% de las portadoras de la premutación, tienen desaparición de sangrado menstrual por un año antes de los 40 años, lo que hace un diagnóstico de falla ovárica prematura; esta frecuencia esta aumentada 20 veces al compararlo con la población general, en la que la falla ovárica prematura esta alrededor del 1% 14 . Además, se ha descrito que la falla ovárica esta aumentada en las portadoras en todas las edades en que se han logrado desarrollar estudios, a los 18 años 1.4%, a los 29 años 3.0% (en la población general 1 en 1,000), y tienen menopausia a más temprana edad después de los 40 años 14. En estas mujeres se encuentran signos y síntomas secundarios como alteración hormonal con niveles de FSH elevados y niveles de estradiol bajos, ciclos menstruales irregulares, síntomas perimenopáusicos y disminución de la reproducción 2, 8, 15, 16. 

En la paciente aquí reportada la asociación de ciclos menstruales irregulares que obligaron a la histerectomía a los 33 años, síntomas vagales (relacionados con menopausia) y el hallazgo de premutación del gen FMR1, nos llevaron a concluir que la paciente tuvo falla ovárica prematura y FXPOI.

El FXTAS es un desorden neurodegenerativo progresivo caracterizado por déficits neurológicos que incluyen temblor progresivo de intención, ataxia cerebelar, déficit cognitivo, parkinsonismo, neuropatías, disfunción autonómica y demencia; este síndrome se presentan en el 40% de los hombres portadores de la premutación a los 60 años y en el 75% a los 80 años, y en el 20% de las mujeres 3, 4, 9 . En la población general la prevalencia estimada es de 1 en 4,000 hombres después de los 55 años y de 1 en 7,800 en mujeres 3

En el FXTAS la alteración en el gen FMR1 no produce el silenciamiento del gen FMR1 como en los afectados por el SXF en quienes la FMRP (Fragile X Mental Retardation Protein) esta disminuida o ausente 3,17, por el contrario hay una sobre expresión, produciendo entre 2-8 veces la cantidad normal de mRNA del FMR1 2,3,4, este último sería el responsable de la toxicidad neuronal y llevaría a la presentación tardía de desórdenes neurodegenerativos 4. En el FXPOI no se ha logrado demostrar como la elevación del mRNA afecta el eje hipotálamo-hipófisis-gónada o al ovario directamente 16. 

El diagnóstico de FXTAS en portadores de la premutación del gen FMR1 combina criterios clínicos, radiológicos de neuroimágenes y patológicos 12. Los criterios mayores son temblor de intención, ataxia cerebelar, deterioro cognitivo e hiperintensidad del pedúnculo cerebelar medio e inclusiones intranucleares redondeadas típicas de FXTAS en neuronas y astrocitos; los criterios menores incluyen problemas de memoria a corto plazo, de intensidad moderada-severa, neuropatía, déficit en las funciones ejecutivas, parkinsonismo, atrofia cerebral generalizada, enfermedad de la materia blanca en cerebro o en cerebelo. Los síntomas son menos severos en las mujeres 3,4, 18.

Para un diagnóstico definitivo del FXTAS es necesario tener dos criterios mayores: un criterio clínico mayor más uno radiológico mayor o uno patológico; la presencia de dos criterios clínicos mayores o un criterio clínico menor más un criterio radiológico menor hacen un diagnostico probable. Por último un diagnóstico posible de FXTAS se constituye a partir de la presencia de un criterio clínico menor y un criterio clínico menor 3,4.

El diagnóstico del FXTAS en la paciente aquí reportada se hizo por tener dos criterios clínicos mayores: temblor de intensión severo y deterioro cognitivo sustentado en un puntaje menor al esperado en el test de MINIMENTAL; y el criterio radiológico menor atrofia cortical generalizada y marcada en la región frontal; además, presentó ansiedad generalizada y neuropatía por síndrome del túnel carpiano. La severidad de los síntomas y la edad temprana aparición del FXTAS hacen que el caso sea poco frecuente y de mal pronóstico.

Sin embargo, en esta paciente la edad de aparición de FXPOI alrededor de los 33 años, la edad de inicio de síntomas del FXTAS antes de los 50 años, la severidad de los síntomas como temblor de intensión que le impide realizar actividades de la vida diaria, temblor mandibular, déficit cognitivo, ansiedad y atrofia cortical generalizada nos hace considerar que se trata de un caso muy poco frecuente, no solo por la baja prevalencia del FXTAS en mujeres, sino también por la escasa presentación de FXTAS y FXPOI simultáneamente, con una historia natural de la enfermedad inusual. 

El tratamiento en el FXTAS es principalmente sintomático con opciones terapéuticas que buscan aliviar los síntomas neuromotores y psiquiátricos, siendo los beta bloqueadores, como el propanolol, los más usados para el alivio del temblor esencial, seguidos de los anticonvulsivantes. Pacientes con ataxia han mostrado mejoría con el uso de memantina 19. Los inhibidores selectivos de la recaptación de serotonina (IRSS) indicados en muchos de los trastornos mixtos, asociados a ansiedad o depresión, son de utilidad en los pacientes con FXTAS; así como lo son la actividad física, las actividades lúdicas, el suplemento de folatos y vitamina B12, son usados con el objetivo de mantener las capacidades motoras, cognitivas y disminuir la atrofia cerebral 3,19. La descripción de las FRAXOPATIAS 4 ha sido relativamente reciente y el acceso a pruebas moleculares es limitado en nuestro medio. Sugerimos que en pacientes con falla ovárica prematura, temblor y/o ataxia se debe sospechar que son portadores premutación del gen FMR1, la probabilidad del diagnóstico se aumenta cuando existen antecedentes familiares específicos de SXF o discapacidad intelectual de origen desconocido. También, cuando se encuentran en las imágenes cerebrales los criterios específicos. El diagnóstico se debe descartar o confirmar con pruebas moleculares como PCR y Southern Blot específicos para el gen FMR1 2.

## Conclusiones

Se aporta un paciente con criterios clínicos y radiológicos de FXTAS y FXPOI con aparición de los síntomas a edad temprana y sintomatología severa, mostrando una historia natural de la enfermedad poco frecuente en portadoras de la premutación del gen FMR1
